# Development of Children’s monitoring and control when learning from texts: effects of age and test format

**DOI:** 10.1007/s11409-019-09208-5

**Published:** 2019-09-07

**Authors:** Martina Steiner, Mariëtte H. van Loon, Natalie S. Bayard, Claudia M. Roebers

**Affiliations:** Institute of Psychology, Department of Developmental Psychology, University of Bern, Fabrikstrasse 8, 3012 Bern, Switzerland

**Keywords:** Monitoring, Control, Development, Test format, Stability

## Abstract

This study investigated elementary school children’s development of monitoring and control when learning from texts. Second (*N* = 138) and fourth (*N* = 164) graders were tested in the middle (T_1_) and end (T_2_) of the school year. The study focused on the cross-sectional and longitudinal development of monitoring and control, and aimed to investigate the development of metacognition for two test formats. After reading expository texts, children completed a comprehension test consisting of open-ended and true-false questions. They monitored their test performance by making confidence judgments, and controlled performance by deciding whether to maintain or withdraw their given answers. Overall, monitoring and control accuracy was higher for open-ended questions than for true-false questions. For open-ended questions, results indicated higher metacognitive accuracy for fourth graders than second graders. No such age effects were found for monitoring and control for true-false questions. Longitudinally, children of both age groups improved their monitoring and control accuracy from T_1_ to T_2_, for open-ended and true-false questions. For both test types, improvement mainly occurred for the monitoring and controlling of incorrect, rather than correct answers. Additionally, the results indicated inter-individual stability of performance, but no stability of monitoring and control accuracy over time. The findings indicate that developmental as well as task-related factors affect children’s metacognitive accuracy.

**Electronic supplementary material:**

The online version of this article (10.1007/s11409-019-09208-5) contains supplementary material, which is available to authorized users.

Learning from texts and adequately retrieving and reproducing that information when answering questions during test taking is important in daily school life. Effective learning and test taking behavior are characterized by accurate *monitoring* (i.e., evaluation of ongoing mental activities such as confidence in the correctness of an answer) and *control* (i.e., regulation of ongoing mental activities such as restudying information or withdrawing answers) (also referred to as “metacognition”; Nelson and Narens 1990). Accurate monitoring and adaptive control are positively related to performance (Dunlosky and Rawson 2012). Thus, inaccurate monitoring and control can hinder successful performance and the effectiveness of subsequent study behavior (Little and McDaniel 2015). Previous research has shown that younger elementary school children’s monitoring and control is often inaccurate; they tend to overestimate their performance (Schneider and Löffler 2016). However, although learning from texts and retrieving the studied information is important for learning in elementary school, very few studies have examined the developmental progression in metacognitive skills when young children monitor and control their comprehension of texts during test taking. The present study aims to fill that gap by investigating the development of children’s metacognition when taking text comprehension tests.

For testing text comprehension in school, different test formats are used (Magliano et al. 2007). Research with adults has shown that depending on the test format, the accuracy of monitoring and control can substantially differ, with open-ended recall questions being typically more accurately monitored and controlled than forced-choice recognition questions (Thiede and Dunlosky 1994). With the present study, besides investigating elementary school students’ development of monitoring and control when completing a text comprehension test, we also aim to investigate the effect of test format on their metacognitive accuracy. Understanding how the design of a test affects students’ monitoring and control may contribute to creating test conditions that support children to make accurate judgments, and thereby improving their performance.

## Development of monitoring and control during test taking

Metacognitive monitoring and control skills undergo continuous improvements during childhood, especially over the elementary school years (for a review, see Schneider and Löffler 2016). Younger elementary school children tend to overestimate their learning, which has detrimental effects on their regulation of study and performance: They tend to terminate study time prematurely, fail to restudy material that needs more repetition, or maintain incorrect answers, which consequently causes lower achievement (Dufresne and Kobasigawa 1989; Roebers et al. 2009; Van Loon et al. 2017).

Ideally, when monitoring the accuracy of one’s test answers, one would be more confident for correct answers than for incorrect answers (“discrimination”; Dunlosky and Thiede 2013). Koriat and Ackerman (2010) found that older elementary school children (11 yrs.) discriminated more between correct and incorrect answers than younger elementary school children (8/9 yrs.) when giving confidence judgments (CJs) for their answers in a general knowledge test. Similar results were obtained by Pressley et al. (1987), who compared older (9–11 yrs.) and younger (6–8 yrs.) children’s discrimination ability when monitoring their answers in a picture vocabulary test. When looking at improvement in discrimination ability in more detail, findings indicate that it is mainly children’s confidence for incorrect answers that changes over time. When judging incorrect responses, young students are often overconfident. Hence, CJs for incorrect answers are rather high in younger elementary school students, but decrease throughout the following school years (Krebs and Roebers 2010; Roderer and Roebers 2010). Not only monitoring skills, but also children’s ability to control their test taking behavior develops during these years. When having the option to withdraw answers from a test to raise the quality of test performance, older elementary school students more likely withdraw their incorrect answers, whereas younger elementary school students tend to maintain a substantial amount of their incorrect answers (Koriat and Ackerman 2010; Krebs and Roebers 2010).

A main reason for children’s improvement in their monitoring accuracy is that they increasingly use valid cues when judging their performance (Koriat and Ackerman 2010). This relates to the theory that monitoring judgments are based on certain cues that are more or less indicative of actual performance accuracy (i.e., “cue validity”; Koriat 1997). Younger children have a tendency to base their monitoring judgments on motivational factors, such as wishful thinking or the effort they put in a task (Schneider 1998). This does not necessary reflect their actual performance, and results in inappropriately high confidence for incorrect answers. However, with increasing age, children learn to base their monitoring judgments on cues that are more reflective of their performance, such as retrieval fluency (Koriat and Ackerman 2010; Roebers et al. 2019) or accessibility (Van Loon et al. 2017). That is, older children more likely take the ease of retrieving an answer into account when giving CJs for their answers. Those improvements in monitoring accuracy ultimately lead to improvements in controlling one’s test performance, as control relies on monitoring judgments (“monitoring-based control”; Metcalfe and Finn 2013). Even though younger elementary school children are able to relate their control decisions to their monitoring judgments, this relationship between monitoring and control, i.e., the tendency to maintain answers with high confidence and withdraw answers with low confidence, becomes even more consistent throughout elementary school (Hembacher and Ghetti 2013; Koriat and Ackerman 2010).

Improvements in cue utilization or monitoring-based control during early school years are likely to be fueled by children’s brain maturation and experiences at school. During elementary school years, the prefrontal cortex, which has been associated with metacognitive accuracy (Fleming and Dolan 2012; Pannu and Kaszniak 2005), undergoes substantial changes (Diamond 2002). These changes encompass aspects such as speed of processing or the ability to use strategies related to higher cognitive functions such as metacognition (Diamond 2002). On the other hand, as soon as children enter school, they are exposed to a stimulating environment, that causes improvements in metacognitive abilities. Research points out possible impacts of teachers on children’s metacognitive skills, e.g., by giving strategy suggestions, asking questions that foster metacognition, or giving adequate feedback (Coffman et al. 2008; Grammer et al. 2013; Llorens et al. 2014; Van Loon and Roebers 2017). In sum, literature suggests that due to brain maturation and environmental influences, children improve their monitoring and control skills throughout elementary school.

## Stability of monitoring and control

Findings about elementary school children’s monitoring and control accuracy mostly stem from studies that measured metacognition cross-sectionally. However, as Dunlosky and Thiede (2013) emphasize, understanding fluctuations in monitoring judgments and monitoring accuracy over time is essential to foster a consistently high accuracy of students’ monitoring and control. Fluctuations can be investigated by measuring the stability of monitoring accuracy over a certain time period. Stability has typically been investigated by correlating learners’ metacognitive accuracy from two or more time points (e.g., Jonsson and Allwood 2003; Stankov and Crawford 1996; Thompson and Mason 1996). Only very few studies focused on elementary school children’s stability of metacognitive accuracy. Roebers and Spiess (2017) measured 8−/9-year olds’ stability of monitoring accuracy and control accuracy twice within 8 months when children completed a spelling task. They found that, whereas stability of performance was high, stability of monitoring and control accuracy was low. This means that a student who, relatively to his or her classmates, discriminated well between correct and incorrect answers at one time point, does not necessarily discriminate well the next time. Further evidence comes from Buratti et al. (2014), who found instability of discrimination ability in elementary school children as well as adults when measurement points were separated by one week. In sum, these few studies indicate that stability of monitoring and control in elementary school students may be low. However, it is yet unknown whether this is also the case when children monitor and control their text comprehension.

## Monitoring and control of text comprehension

Most studies addressing metacognition in elementary school children have been conducted using associative learning tasks such as learning word pairs or picture-word pairs (Thiede et al. 2009). However, in the educational context, students often need to comprehend and memorize information that is presented in texts (Moss 2005). Several studies focused on students’ comprehension monitoring *during reading* by investigating their ability to detect inconsistent information in a text, or predict test performance (August et al. 1984; Cain et al. 2004; De Bruin et al. 2011; Markman 1979; Van Loon et al. 2014; Vidal-Abarca et al. 2010). Generally, findings indicate that accurately monitoring one’s comprehension during reading is challenging for children, and remains difficult, even for adults (De Bruin et al. 2011; Markman 1979; Thiede et al. 2009). This monitoring inaccuracy during reading likely results in inadequate control, because control of reading processes, such as restudying texts, relies on monitoring during reading (De Bruin et al. 2011; Van Loon et al. 2014; Vidal-Abarca et al. 2010). In contrast to research efforts investigating metacognition during reading, empirical evidence on children’s monitoring and controlling during retrieval of textual information, i.e., when taking a comprehension test, is limited.

Accurate monitoring *during retrieval* of textual information when taking a test enables learners to diagnose what they know and what they don’t know, which ideally, improves the effectiveness of subsequent study (Little and McDaniel 2015; Thiede et al. 2017). Thiede et al. (2017) investigated how different strategies of subsequent study after taking a test affects further performance. They let secondary school students read three texts and complete a text comprehension test. After that, to increase overall performance on a final test, students either picked one of the texts for rereading themselves, or they were automatically presented with the text for which they had the lowest performance. The group of students who reread the text with the lowest initial performance could significantly improve their performance on the final text comprehension test, whereas those who reread the self-picked text only marginally improved. This emphasizes the impact of adequate control of study on performance, but at the same time shows that adequate control is challenging, even for older students. However, evidence suggests that secondary school students’ control of study in text comprehension tests can be improved by fostering children’s metacognitive skills during the early years of their school careers. Exposing elementary school children to text comprehension tests that emphasize deep comprehension, seems relevant for their control of study in later school years (Thiede et al. 2012).

## Effects of question format on monitoring and control

Text comprehension is usually assessed with a series of questions about the text. The most common formats are open-ended recall questions and recognition questions in a multiple-choice or true-false answer format (Magliano et al. 2007; Thiede and Dunlosky 1994). Importantly, when judging the correctness of one’s answers, monitoring accuracy seems to be affected by test format. Thiede and Dunlosky (1994) showed that university students’ monitoring accuracy in a paired associate task was higher when judging recall performance than when judging recognition. The same result was found when students were tested with short-answer questions and multiple-choice questions about the content of a course (De Carvalho Filho 2009). As test format has an impact on monitoring of test performance, it is not surprising that control seems to be affected too. When adult students were given the option to reread texts in order to revise and improve their answers, they made more appropriate rereading decisions after completing a short-answer open-ended test than after completing multiple-choice test questions (Pressley et al. 1990). Similar results were obtained by Ferrer et al. (2017), who gave one group of high school students the option to reread texts during answering questions. Students were more likely to reread texts during answering open-ended questions compared to multiple-choice questions. Rereading decisions during answering open-ended questions were effective, as it helped students to improve performance, compared to the group who was not given the option to reread. No such advantage in performance was observed for students who had the option to reread texts during answering multiple-choice questions compared to students who did not have this option. Thus, results indicate that controlling one’s performance during answering multiple-choice questions may be less efficient than during answering open-ended questions.

Empirical evidence concerning the effects of test format on children’s monitoring and control is limited. Roebers and Howie (2003) tested elementary school students about the content of a short video by using open-ended questions (e.g., “How did the boys get to the farmhouse?”) as well as so-called “misleading questions”—true-false questions that suggested an incorrect answer (e.g., “The boys came by car to the farmhouse, right?”). Students’ monitoring accuracy was affected by test format; for open-ended questions, children made accurate monitoring judgments by indicating higher confidence for correct compared to incorrect answers. In contrast, monitoring was inaccurate for misleading true-false questions, that is, confidence was at least as high for incorrect answers as for correct answers. However, it should be noted that the misleading questions format may not be common in the school setting, and possibly, children’s inaccurate monitoring may be due to the unusual format of these questions.

Differences in monitoring and control accuracy between recall and recognition questions can be explained in terms of the quality and quantity of monitoring cues available during answering. When responding to recall questions, one needs to actively recollect information from memory (Yonelinas 2002). Trying to retrieve information from memory may provide the test taker with insight about the accessibility or the retrieval fluency of the information, which is related to the quality of performance (Koriat and Ackerman 2010; Van Loon et al. 2017). That is, when an individual answers an open-ended question easily and fast, that answer is more likely to be correct, and should therefore result in a high CJ, whereas problems with retrieving information should result in a lower CJ, since the quality of the answer is likely to be poorer. When answering recognition questions (e.g., multiple-choice or true-false questions), on the other hand, an individual more likely relies on stimulus familiarity than on active information recollection from memory. That is, because the target information is present in the answer options the question can be answered with the aid of perceived familiarity (Yonelinas 2002). This perceived familiarity may provide the test taker with less valid cues about the correctness of the answer, and can lead to erroneously high CJs for incorrect answers.

In sum, studies with adults suggest more accurate monitoring and control when completing recall tests, rather than recognition tests. However, for children, effects of test format on metacognition are yet unclear. In the present study, we therefore investigated children’s monitoring and control with open-ended recall and true-false recognition questions, and we explored whether children’s developmental improvement in monitoring and control skills over time differs between the two test formats.

## Present study

Previous research mainly addressed improvements in monitoring and control during elementary school years by comparing different age groups in the context of simple learning tasks. The aim of the present study is to investigate if such improvements similarly occur when solving a more complex text comprehension test. To address this question, we tested second and fourth graders twice within an interval of 6 months, which allowed us to compare metacognitive accuracy cross-sectionally between two age groups, as well as investigate development longitudinally between the first and the second measurement point. This approach is also encouraged by Winne and Nesbit (2010), who recommend that changes in metacognitive ability over time should be assessed by a combination of a cross-sectional and longitudinal design.

Previous research with simple learning tasks showed that older elementary school children more accurately monitor and control their test performance than younger elementary school children (e.g., Koriat and Ackerman 2010). Based on these findings, we expected improvements over the elementary school years in monitoring and control when engaging in a text comprehension task, especially for incorrect answers (e.g., Krebs and Roebers 2010). Specifically, we addressed the following hypotheses:

1. Fourth graders show more accurate monitoring and control skills and more monitoring-based control than second graders (*Effect of age; cross-sectional*).
2. Monitoring, control and monitoring-based control abilities improve from the first (T_1_) to the second (T_2_) measurement point for second as well as for fourth graders (*Effect of time; longitudinal*).

Even though performance seems to be stable over time, previous research indicates that monitoring and control accuracy is rather instable (Buratti et al. 2014; Roebers and Spiess 2017). Therefore, we expected the following:

3. Stability of monitoring and control is low for second and fourth graders.

The above-mentioned hypotheses were tested for open-ended and true-false questions to investigate the differences between the two test formats. In line with research that investigated difference in monitoring and control accuracy when answering recall compared to recognition questions (e.g., Ferrer et al. 2017; Thiede and Dunlosky 1994), we tested the following hypothesis:

4. Second as well as fourth graders monitor and control answers to open-ended questions more accurately than answers to true-false questions.

## Method

### Participants

The sample consisted of 327 children at T_1_, and 324 children at T_2_, from 21 classes. Class sizes (including the children who did not receive informed consent to participate) varied between 16 and 24 children, which corresponds to standard class sizes in the study’s country. Schools were selected by contacting principals from public schools in the larger vicinity of a mid-sized university town. Family backgrounds were lower to upper middle class. For the analyses, age outliers were excluded (*N* = 19). Further, sample attrition (*N* = 6) was due to incomplete test completion, insufficient language skills, eye problems when reading on a tablet, and change in residence. The data of 302 children were used for analyses to test our hypotheses (*n* = 138 second graders; 50% female; *M* age = 7.6 years, *SD* = .5 years, and *n* = 164 fourth graders; 47% female; *M* age = 9.6 years, *SD* = .5 years). Sixty-five children (21.5%) were non-native speakers. Outcomes related to our hypotheses did not differ for non-native speakers, indicating that they had sufficient language skills to follow the instructions. Parental informed consent was received prior to testing. Children gave oral consent prior to the test sessions and were informed that they could withdraw participation at any time without consequences. No child ever did.

### Pilot study

Prior to the actual study, we conducted a pilot study in order to select two subsets (one subset for each measurement point) of six texts with four questions each, that cover a comparable range of easy, medium and difficult questions. That range in difficulty had to be as similar as possible not only for T_1_ and T_2_, but also for second and fourth graders. Therefore, we piloted 18 texts with subsets of two open-ended questions and three true-false questions per text. The sample of the pilot study consisted of one second (*N* = 20) and one fourth grade classroom (*N* = 15), with children in the same age as children at T_2_. The texts and questions we used in this pilot study were translated and adapted from studies by De Bruin et al. (2011) and Van Loon et al. (2015). Texts for fourth graders were longer and more complex, and questions were more difficult for fourth graders than for second graders.

The goal of the pilot study was to select two subsets of six texts with two open-ended and two true-false questions per text that met the following criteria: First, text-question subsets that elicited ceiling or bottom effects in one of the open-ended questions or in more than one of the true-false questions were removed. We expected greater ceiling effects for true-false questions. Therefore, piloting three true-false questions allowed us to exclude one from each text-question set without removing the whole text-questions subset from the sample. Additionally, each sample of true-false questions at both measurement points included 6 true-false questions that were correct and 6 questions that were incorrect. Second, to ensure a suitable range of variance in question difficulty, we chose text-question sets that provided together a similar amount of easy (~ 30%), medium (~ 40%) and difficult (~ 30%) open-ended and true-false questions, for both age groups at both measurement points. And third, to make a comparison between the two age groups most reliable, for both age groups the same subsets of 12 texts (in a difficult and in an easier form) were chosen.

The mean length of the 12 selected texts was 87 words for second graders and 126 words for fourth graders. The complexity of the texts as measured by the LIX (readability index; see Björnsson 1983) was 31.53 for second graders and 37.81 for fourth graders, respectively, indicating that the readability ranged between easy and moderate.

### Materials and procedure

Each class was visited by four experimenters. At both measurement points the classes were split into two groups of 6 to 11 children, and each group was then tested in an individual room with two experimenters present. Shortly after completing the first measurement point in all classes, teachers were contacted by email to schedule a date for the second measurement point (number of days between the two measurement points: *M* = 181.6, *SD* = 13.8). Prior to completing the task, children were instructed about the whole task procedure (reading, answering questions, giving CJs, and maintaining/withdrawing questions) by one of the experimenters. This instruction took place during the small-groups session, after separating the class in two groups. There were no more additional instructions given during the task, however, children could read instructions for each step and instructions were repeated for individual children if needed. With practical and interactive examples, the experimenters ensured that children understood the task.

**Fig. 1:**
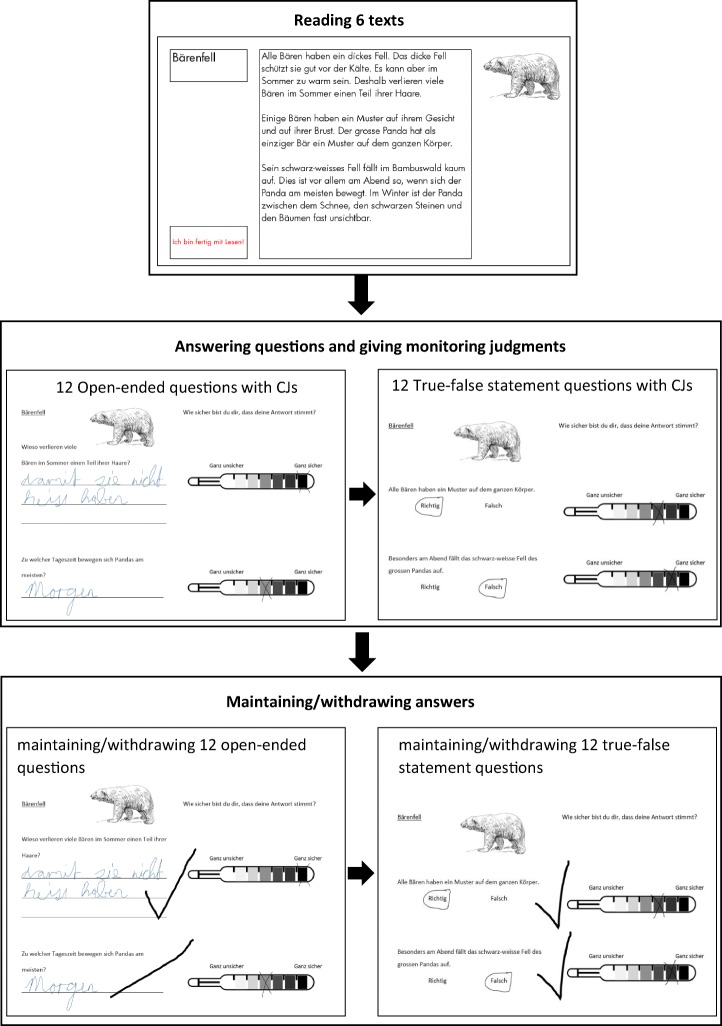
Schematic task procedure. CJs = confidence judgments

The text comprehension task included the following phases: 1) study (text reading)^1^; 2) test about text content with two question formats; 3) monitoring (CJs); and 4) control (selection of answers). Figure 1 shows the phases of the task that are relevant for our study.

#### Study

For the study phase, students read six expository texts on a tablet. Presenting the texts on a tablet enabled a complete randomization of the texts. Further, it standardized the reading procedure for children by not allowing them to move back and forth between texts. The study phase was self-paced; however, the minimum reading duration before having the option to move on to the next text was 10s, ensuring that children could not click through. The computer displays were 11.6 “monitors with a resolution of 1366 × 768 pixels. The text font was Futura Std. Book and the size was 30 for second graders and 25 for fourth graders. Complete texts were presented on screens; no scrolling was needed. The screen for each text contained the title of the text, the text, and a content-related picture. Topics were animals (“Bees”, “Bears”, “Dragonflies”, “Camels“), geographical/ geological/ meteorological subjects (“Tropics”, “Desert”, “Egypt”, “Nile”, “Seasons”, “Stars“), or physiological processes (“Cold”, “Chewing gum”). An example of a text is presented in Appendix A.

#### Test

The text comprehension test consisted of twelve open-ended and twelve true-false questions (two open-ended and two true-false questions per text) presented in a booklet (for examples of questions see Appendix A). The first part of the test consisted of 12 open-ended questions and the second part of 12 true-false questions. Ozuru et al. (2013) showed that presenting open-ended recall questions before recognition questions did not have an effect on performance of recognition questions. The two open-ended questions that belonged to the same text were always presented together on one page, and the same applied to the true-false questions. Participants were encouraged to answer all open-ended questions; however, they were allowed to put a question mark below an open-ended question if they could not think of any answer. As answers to open-ended questions typically range from a single word to one or more sentences (Magliano et al. 2007), we included two types of open-ended questions in our study to fully represent that question format. One of the two open-ended questions per text required a single word (detail question) and the other required a sentence (causal question). When answering true-false questions, participants had to choose either “true” or “false.” The order of topics was counterbalanced. Internal consistency for the 12 open-ended questions was Cronbach’s α = .70 at T_1_ and .60 at T_2_ for second graders, and .68 on T_1_ and .63 at T_2_ for fourth graders. Internal consistency for the 12 true-false questions was Cronbach’s α = .37 at T_1_ and .16 at T_2_ for second graders, and .45 on T_1_ and .20 at T_2_ for fourth graders. Internal consistency of the true-false questions is rather low, however, low internal consistency is not uncommon for text comprehension tests (cf. Engelen et al. 2018; Van Loon et al. 2015). It lies in the very nature of metacognitive research that questions for which monitoring judgments are gathered, cover the entire variation of difficulty to allow tapping the whole range of monitoring processes. Furthermore, while internal consistency is low, both, open-ended and true-false question tests appear to be measuring similar knowledge about the texts, as shown by moderate to high correlations between open-end and true-false tests at both measurement points and for both grades (*r*s *=* ≥ .41, *p*s < .001).

#### Monitoring

Immediately after giving each answer, children were requested to make a CJ by indicating how certain they were that their answer was correct. For that purpose, a 7-point Likert scale was depicted to the right of each question. The scale had the shape of a thermometer, that ranged from white (*very unsure*) to black (*very sure*), with different shades of grey in the middle (adapted from Koriat and Shitzer-Reichert 2002).

#### Control

After answering all questions and making all CJs, children were instructed to go through their answers again and decide whether they wanted to maintain or withdraw them. For that purpose, children could add a check mark behind an answer to maintain the answer; to withdraw, they could cross out the answer. In the introduction, prior to starting the task, children were told that this answer selection procedure was a competition between the participating classes, and that they could gain or lose points with this task. The bonus-to-penalty-ratio by Roebers et al. (2009) was used as it has been shown to motivate children to control their performance. Maintaining a correct answer was rewarded with 1 point; however, maintaining an incorrect answer caused a loss of 3 points. Withdrawing an answer gave 0 points, independent of its correctness. For ethical and motivational reasons, this was a mock competition and all classes received a gift after participation.

### Dependent measures and analyses

To assess performance, answers to true-false and open-ended questions were scored as correct (1) or incorrect (0). In accordance with Van Loon et al. (2015), comprehension was emphasized when scoring open-ended questions. Therefore, verbatim responses showing the literal text content as well as gist responses showing that children understood the meaning were both scored as correct. Two independent raters coded 100% of the answers for open-ended questions at both measurement points. Interrater reliability was very high at T_1_ (Kappa = .95, *p* < .001) and T_2_ (Kappa = .89, *p* < .001).

To assess monitoring accuracy, mean CJs for correct and incorrect answers were compared to see whether children could discriminate between their correct and incorrect responses (cf. Destan et al. 2014; Dunlosky and Thiede 2013). Aggregated data, such as mean CJs for correct and incorrect answers, is typically used in the context of discrimination (Schraw 2009). Similarly, control accuracy was measured by the mean percentage of answers maintained for correct and incorrect answers. Control is effective when a child maintains a high number of correct answers and a low number of incorrect answers. Repeated measures ANOVAs with correctness (correct vs. incorrect answer) and measurement point (T_1_ vs. T_2_) as within-subject factors and age (second vs. fourth grade) as a between-subjects factor were conducted for mean CJs and mean percentages of correct and incorrect answers maintained.

To further interpret monitoring accuracy, we calculated intra-individual gamma correlations (cf. Nelson 1984) between the CJs and performance (correct vs. incorrect), separately for the set of 12 open-ended and the set of 12 true-false questions. For control accuracy, we calculated intra-individual phi correlations between withdrawal decisions (maintain vs. withdraw) and performance, separately the set of 12 open-ended and the set of 12 true-false questions. We used phi correlations because withdrawal decision and performance are both binary variables (Chedzoy 2006). For both, gamma correlations as well as phi correlations values closer to 1 indicate higher monitoring/control accuracy, whereas values closer to 0 indicate low monitoring/control accuracy. Gamma correlations were also used to investigate the relation between CJs and control. For gamma and phi correlations, repeated measures ANOVAs with measurement point (T_1_ vs. T_2_) as a within-subject factor and age (second vs. fourth grade) as a between-subjects factor were conducted to investigate whether there were effects of measurement point and age on monitoring and control. Stability between T_1_ and T_2_ was assessed with Pearson correlations for performance (percentage of correct answers) and accuracy of monitoring and control (gamma and phi correlations). To investigate the effects of test format, all analyses were conducted separately for both test formats (cf. Roebers 2006). Significant interaction effects were assessed with either repeated measures ANOVAs or paired sample T-tests.

Only completed test items were included in the analyses. In line with Roebers et al. (2007), question marks were coded as omissions. These omissions were not included in further analyses because children did not make CJs for these, and did not decide on maintaining or withdrawing. The percentage of omissions for open-ended questions is reported in Table 1 (no omissions for true-false questions). The percentage of missings (no answer, no question mark) was low, both for open-ended questions (*M* = 2.37%) and true-false questions (*M* = .19%).

**Table 1:** Mean performance, CJ, and percentage of answers maintained

	2nd grade		4th grade	
	T_1_	T_2_	T_1_	T_2_
Open-ended questions				
% correct answers	42.75 *(23.31)*	52.48 *(19.84)*	49.28 *(22.06)*	50.21 *(19.53)*
% incorrect answers	33.70 *(18.91)*	35.87 *(18.77)*	35.63 *(20.42)*	36.57 *(20.38)*
% omissions	17.81 *(18.98)*	8.64 *(13.71)*	14.62 *(15.13)*	12.96 *(11.56)*
CJ	5.11 *(1.32)*	4.87 *(1.19)*	4.70 *(1.24)*	4.56 *(1.21)*
% answers maintained	72.38 *(24.78)*	65.52 *(22.83)*	60.07 *(23.19)*	58.06 *(20.68)*
True-false questions				
% correct answers	57.73 *(16.38)*	68.84 *(16.49)*	56.95 *(15.19)*	64.02 *(13.59)*
CJ	5.29 *(1.22)*	5.30 *(1.13)*	5.21 *(1.15)*	5.29 *(1.11)*
% answers maintained	71.73 *(25.24)*	70.07 *(22.01)*	66.37 *(23.24)*	69.11 *(21.86)*

*Note*. Standard deviations in parentheses

## Results

In this section, we report the analyses testing the hypothesized effects of age group, measurement point and test format. Before reporting findings on accuracy and stability of monitoring and control, we report preliminary results on test performance for the two test formats.

### Test performance

To investigate differences in performance between age groups and measurement points, we conducted repeated measures ANOVAs for both test formats separately. Table 1 shows the percentage of correct responses for both test formats, as well as incorrect answers and omissions for open-ended questions.

#### Open-ended questions

For open-ended questions, children had more correct answers at T_2_ compared to T_1_, *F* (1, 297) = 23.73, *p* < .001, η_p_^2^ = .07, whereas no difference was found between second and fourth graders (*p* = .35). The significant interaction between measurement point and age, *F* (1, 297) = 12.73, *p* < .001, η_p_^2^ = .04, showed that second graders’ increased their performance from T_1_ to T_2_, *F* (1, 137), *p* < .001, η_p_^2^ = .17, whereas fourth graders’ performance did not change (*p* = .295). For the number of incorrect answers, no differences were found between T_1_ and T_2_, and between second and fourth graders (*p*s ≥ .32). Children had fewer omission errors at T_2_ compared to T_1_, *F* (1, 297) = 28.01, *p* < .001, η_p_^2^ = .09, whereas no difference was found between second and fourth graders (*p* = .63). The significant interaction between measurement point and age, *F* (1, 297) = 13.19, *p* < .001, η_p_^2^ = .04, revealed that second graders reduced the number of omission errors from T_1_ to T_2,_*F* (1, 137) = 28.02, *p* < .001, η_p_^2^ = .17, whereas fourth graders did not show changes in the number of omissions (*p* = .15).

#### True-false questions

For true-false questions, children had more correct answers at T_2_ compared to T_1_, *F* (1, 299) = 73.18, *p* < .001, η_p_^2^ = .20, whereas no difference was found between second and fourth graders (*p* = .06, η_p_^2^ = .01). Performance for true-false questions was above chance for second graders at T_1_, *t* (137) = 5.54, *p* < .001, Cohen’s *d* = .47, and T_2_, *t* (137) = 13.42, *p* < .001, Cohen’s *d* = 1.14, and for fourth graders at T_1_, *t* (162) = 5.84, *p* < .001, Cohen’s *d* = .46, and T_2_, *t* (163) = 13.22, *p* < .001, Cohen’s *d* = 1.03.

Table 2 shows the stability of performance for open-ended and true-false questions separately for both grades. Performance for open-ended and true-false questions was stable for second and fourth graders. Stability of performance for open-ended questions was significantly higher than for true-false questions, for second graders (Fisher’s *z* = 1.788, *p* = 0.04) as well as fourth graders (Fisher’s *z* = 4.39; *p* < .001).

**Table 2:** Stability of performance, and monitoring and control accuracy (Pearson’s r between T_1_ and T_2_)

	2nd grade	4th grade
Open-ended questions		
Performance	.50**	.62**
Monitoring accuracy	−.16	.00
Control accuracy	.00	.04
True-false questions		
Performance	.32**	.23*
Monitoring accuracy	.00	.17*
Control accuracy	.03	−.01

*Note.* * *p* < .05. ** *p* < .001

### Monitoring

#### Magnitudes of monitoring judgments

To investigate effects of age and measurement point on CJs, repeated measures ANOVAs were conducted for open-ended and true-false questions separately. Table 1 shows the mean CJs for both age groups, measurement points, and test formats. For open-ended questions, children gave lower CJs at T_2_ compared to T_1,_*F* (1, 298) = 7.91, *p* = .005, η_p_^2^ = .03, and fourth graders gave lower CJs compared to second graders, *F* (1, 298) = 8.03, *p* = .005, η_p_^2^ = .03. For true-false questions, there were no significant effects of measurement point and age (*p*s ≥ .47).

#### Accuracy of monitoring judgments

To investigate if monitoring was more accurate for fourth graders than second graders, and more accurate at T_2_ compared to T_1,_ we included correctness (correct vs. incorrect) in the repeated measures ANOVAs.

##### Open-ended questions

**Fig. 2:**
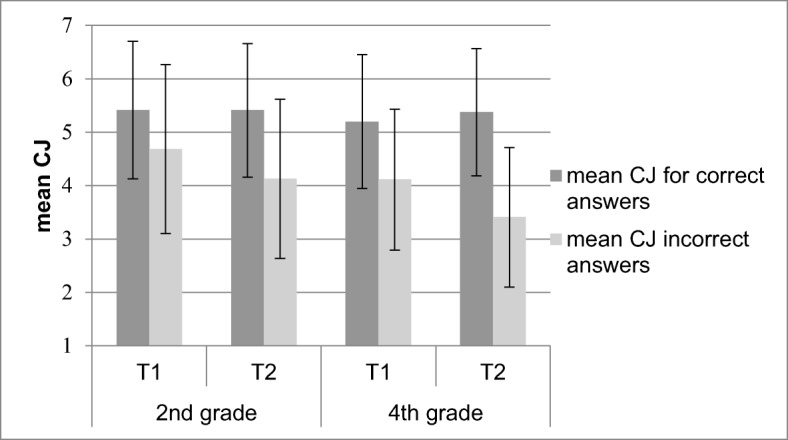
Mean confidence judgment (CJ) for correct and incorrect answers to open-ended questions. Error bars indicate standard deviations

Figure 2 shows the mean CJs for correct and incorrect answers as an effect of age and measurement point for open-ended questions. Overall, children gave lower CJs for incorrect (*M* = 4.08, *SE* = .07) than for correct answers (*M* = 5.35, *SE* = .06), *F* (1, 264) = 461.14, *p* < .001, η_p_^2^ = .64. Consistent with the hypothesis that fourth graders more accurately monitor their incorrect answers than second graders, the interaction between age and correctness was significant, *F* (1, 264) = 19.52, *p* < .001, η_p_^2^ = .07. Fourth graders gave lower CJs for incorrect answers (*M* = 3.76, *SE* = .1) than second graders (*M* = 4.41, *SE* = .1), *F* (1, 264) = 20.48, *p* < .001, η_p_^2^ = .07, whereas CJs for correct answers did not differ between second (*M* = 5.41, *SE* = .1) and fourth graders (*M* = 5.28, *SE* = .1), *p* = .32. Further, in line with the hypothesis that children improve their monitoring of incorrect answers from T_1_ to T_2_, the interaction between measurement point and correctness was significant, *F* (1, 264) = 39.72, *p* < .001, η_p_^2^ = .13. Children gave lower CJs for incorrect answers at T_2_ (*M* = 3.72, *SE* = .09) than at T_1_ (*M* = 4.36, *SE* = .09), *t* (285) = 6.62, *p* < .001, Cohen’s *d* = 0.78, whereas there was no change over time for CJs for correct answers from T_1_ (*M* = 5.31, *SE* = .08) to T_2_ (*M* = 5.42, *SE* = 0.07*), p* = .18. The finding that monitoring of answers to open-ended questions improved for older children and at the second measurement point is also supported by analyses using Gamma correlations, as shown in Table 3. Fourth graders more accurately monitored their performance compared to second graders, *F* (1, 249) = 20.31, *p* < .001, η_p_^2^ = .08, and children more accurately monitored their performance at T_2_ compared to T_1_, *F* (1, 249) = 17.50, *p* < .001, η_p_^2^ = .07.

**Table 3:** Gamma and phi correlations between performance and monitoring, performance and control, and monitoring and control

	2nd grade		4th grade	
	T_1_	T_2_	T_1_	T_2_
Open-ended questions				
Performance – CJs	.27 (.66)	.42 (.54)	.48 (.49)	.65 (.38)
Performance – maintenance	.24 (.39)	.31 (.33)	.28 (.35)	.42 (.32)
CJs – maintenance	.58 (.59)	.72 (.46)	.75 (.42)	.85 (.23)
True-false questions				
Performance – CJs	.07 (.56)	.30 (.58)	.07 (.56)	.30 (.51)
Performance – maintenance	.04 (.33)	.12 (.32)	.10 (.30)	.14 (.31)
CJs – maintenance	.64 (.45)	.72 (.45)	.72 (.46)	.78 (.37)

*Note*. Standard deviations in parentheses

##### True-false questions

**Fig. 3:**
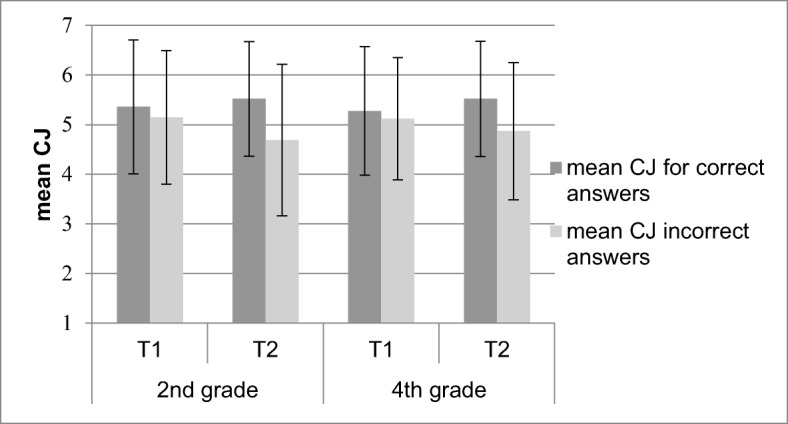
Mean confidence judgment (CJ) for correct and incorrect answers to true-false questions. Error bars indicate standard deviations

Figure 3 shows the mean CJs for correct and incorrect answers as an effect of age and measurement point for true-false questions. Children gave lower CJs for incorrect answers (*M* = 4.96, *SE* = .07) compared to correct answers (*M* = 5.42, *SE* = .06), *F* (1, 293) = 96.16, *p* < .001, η_p_^2^ = .25. No significant interaction was found between age and correctness, indicating that second and fourth graders did not differ in their ability to discriminate between correct and incorrect answers to true-false questions, *F* (1, 293) = 1.55, *p =* .22, *η*_*p*_^*2*^ = .01. However, in line with the hypothesis that children improve their monitoring of incorrect answers from T_1_ to T_2_, the interaction between measurement point and correctness was significant, *F* (1, 293) = 39.57, *p* < .001, η_p_^2^ = .12. Children gave lower CJs for incorrect answers at T_2_ (*M* = 4.79, *SE* = .08) than at T_1_ (*M* = 5.14, *SE* = .07), *t* (295) = 3.77, *p* < .001, Cohen’s *d* = .44, whereas CJs for correct answers were higher at T_2_ (*M* = 5.53, *SE* = .07) than at T_1_ (*M* = 5.31, *SE* = .08), *t* (298) = −2.89, *p* = .004, Cohen’s *d* = .33. As shown in Table 3, the conclusion that monitoring improved between T_1_ and T_2_ was also supported with the gamma correlations, *F* (1, 272) = 21.94, *p* < .001, η_p_^2^ = .08.

##### Open-ended vs. true-false questions

To investigate the differences in monitoring accuracy between open-ended and true-false questions, we conducted repeated measures ANOVAs for T_1_ and T_2_ with test format as a within-subject factor, collapsed across both grades. The difference between the gamma correlations for monitoring accuracy for open-ended and true-false questions was significant at T_1_, *F* (1, 251) = 44.89, *p* < .001, η_p_^2^ = .15, and T_2_, *F* (1, 274) = 35.31, *p* < .001, η_p_^2^ = .11, indicating that monitoring was significantly more accurate for open-ended than for true-false questions at both measurement points.

##### Explorative analyses for detail and causal open-ended questions

Note that open-ended questions either had a detail or a causal focus. To investigate whether there are differences in monitoring accuracy for these two types of open-ended questions (detail/causal), we compared monitoring accuracy for these two question types. Moreover, monitoring for both of these open-ended question types was compared with monitoring for true-false questions. As mentioned above, we found differences between question formats for participants` monitoring of incorrect answers. Because this ability to accurately recognize errors is particularly relevant for improving performance, the following analyses focus on differences in CJs for incorrect answers. Table 4 shows CJs for answers to causal and detail open-ended, as well as true-false questions. Mean CJs for incorrect answers to open-ended questions assessing detail learning were slightly lower than CJs for causal open-ended questions, however, the effect size was small, *t* (280) = 2.59, *p* = .01, Cohen’s *d* = .16. The comparison between CJs for incorrect responses for detail open-ended questions and true-false questions was more pronounced, as indicated by the large effect size, *t* (289) = −11.94, *p* < .001, Cohen’s *d* = .70. Further, a comparison between CJs for incorrect answers to causal open-ended questions and true-false questions also showed a large effect size, *t* (291) = −14.35, *p* < .001, Cohen’s *d* = .88. Thus, even though CJs for incorrect answers were slightly lower for detail than for causal open-ended questions, CJs for incorrect answers to both open-ended question types were considerably lower than CJs for incorrect answers to true-false questions.

**Table 4:** Mean CJs for correct and incorrect answers of causal and detail open-ended questions and true-false questions, collapsed across grade and measurement point

	Causal Open-ended questions	Detail Open-ended Questions	True-False Questions
Correct answers	5.13 (1.31)	5.62 (1.10)	5.43 (1.04)
Incorrect answers	4.09 (1.46)	3.85 (1.43)	4.99 (1.12)

*Note*. Standard deviations in parentheses

##### Stability

Table 2 shows the stability of monitoring accuracy. For open-ended questions, stability both for second and fourth graders was not significant (*p*s ≥ .87). For true-false questions, stability was not significant for second graders (*p* = .99), whereas for fourth graders, stability over time was low but significant, (*p* < .05).

### Control

#### Percentage of answers maintained

To investigate effects of age and measurement point on the percentage of maintained answers, repeated measures ANOVAs were conducted for both test formats separately. Table 1 shows the mean percentage of answers maintained as an effect of age, measurement point, and test format. For open-ended questions, children maintained fewer answers at T_2_ compared to T_1,_*F* (1, 296) = 9.52, *p* = .002, η_p_^2^ = .03, and overall, fourth graders maintained fewer answers than second graders, *F* (1, 296) = 19.65, *p* < .001, η_p_^2^ = .06. For true-false questions, the effects of measurement point and age were not significant (*p* ≥ .19).

#### Accuracy of maintenance

To investigate the hypothesis that control for incorrect answers would be more accurate for fourth graders than second graders, and the hypothesis that control for incorrect answers would be more accurate at T_2_ than at T_1,_ we included correctness (correct/incorrect answers) in the repeated measures ANOVAs.

##### Open-ended questions

**Fig. 4:**
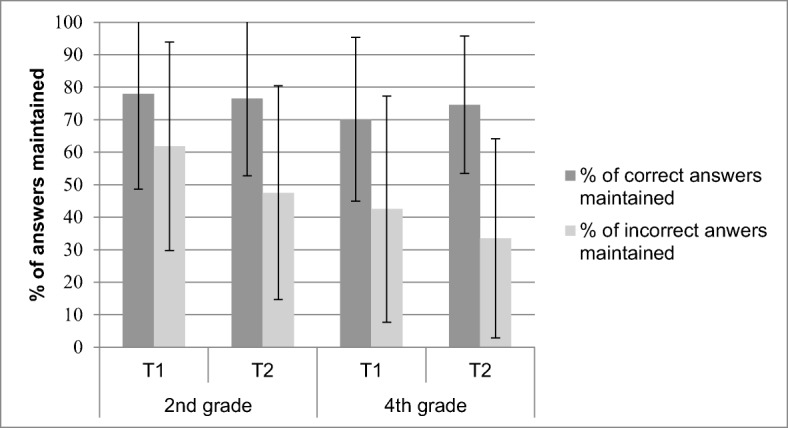
Percentage of correct and incorrect answers maintained for the open-ended questions. Error bars indicate standard deviations

Figure 4 shows the mean percentage of maintained correct and incorrect answers as an effect of age and measurement point for open-ended questions. Overall, children maintained fewer incorrect (*M* = 46.32, *SE* = 1.5) than correct answers (*M* = 74.81, *SE* = 1.16), *F* (1, 263) = 331.6, *p* < .001, η_p_^2^ = .56. Consistent with the hypothesis that fourth graders control their incorrect answers more effectively than second graders, the interaction between age and correctness was significant, *F* (1, 263) = 14.24, *p* < .001, η_p_^2^ = .05. Fourth graders (*M* = 37.96, *SE* = 2.06) maintained fewer incorrect answers than second graders (*M* = 54.68, *SE* = 2.30), *F* (1, 263) = 29.24, *p* < .001, η_p_^2^ = .10, whereas second graders (*M* = 77.26, *SD* = 19.96) maintained more correct answers than fourth graders (*M* = 72.36, *SD* = 17.82), *F* (1, 263) = 4.46, *p* = .036, η_p_^2^ = .02. In line with the hypothesis that control of incorrect answers would be more accurate at T_2_ than T_1_, the interaction between measurement point and correctness was significant, *F* (1, 263) = 20.5, *p* < .001, η_p_^2^ = .07. Children maintained fewer incorrect answers at T_2_ (*M* = 40.40, *SE* = 1.95) than T_1_ (*M* = 51.66, *SE* = 2.10), *t* (277) = 6.62, *p* < .001, Cohen’s *d* = 0.79, whereas the percentage of correct answers maintained did not differ between T_1_ (*M* = 73.94 *SD* = 27.04) and T_2_ (*M* = 76.08, *SD* = 21.94), *p* = .26. The finding that control for open-ended questions improved for older children and at the second measurement point is also supported by phi correlations. As shown in Table 3, fourth graders controlled their performance more accurately than second graders, *F* (1, 211) = 5.17, *p* = .02, η_p_^2^ = .02, at both measurement points, and children controlled their performance more accurately at T_2_ compared to T_1_, *F* (1, 211) = 13.10, *p* < .001, η_p_^2^ = .06, independent of age.

##### True-false questions

**Fig. 5:**
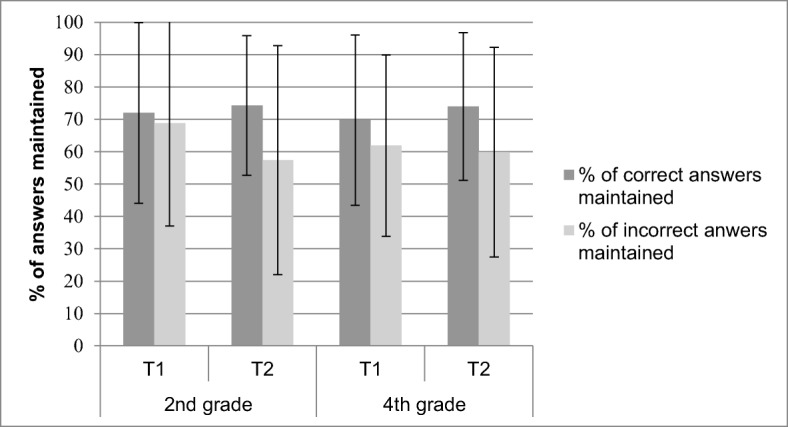
Percentage of correct and incorrect answers maintained for the true-false questions. Error bars indicate standard deviations

For true-false questions, as shown in Fig. 5, children maintained fewer incorrect (*M* = 61.99, *SE* = 1.48) than correct answers (*M* = 72.51, *SE* = 1.17), *F* (1, 293) = 69.86, *p* < .001, η_p_^2^ = .19. Again, no significant interaction was found between age and correctness, indicating that second and fourth graders did not differ in their ability to discriminate between control of correct and incorrect answers to true-false questions, *F* (1, 293) = .14, *p* = .71, η_p_^2^ > .001. However, again, and in line with the hypothesis that control of incorrect answers would be more accurate at T_2_ than T_1_, the interaction between measurement point and correctness was significant, *F* (1, 293) = 17.35, *p* < .001, η_p_^2^ = .06. Children maintained fewer incorrect answers at T_2_ (*M* = 58.77%, *SE* = 1.96) than T_1_ (*M* = 64.97%, *SE* = 1.74), *t* (294) = 2.73, *p* < .001, Cohen’s *d* = .32, whereas they maintained more correct answers at T_2_ (*M* = 74.23, *SE* = 1.28) compared to T_1_ (*M* = 70.86, *SE* = 1.56), *t* (1, 298) = −1.97, *p* = .05, Cohen’s *d* = −.23. As visible in Table 3, the phi correlations further confirm the improvements in control accuracy between T_1_ and T_2_, *F* (1, 218) = 5.59, *p* = .02, η_p_^2^ = .03.

##### Monitoring-based control

To examine the extent to which children related their control decisions to their CJs (monitoring-based control) we calculated gamma correlations between the CJs and the maintenance of answers (see Table 3). As hypothesized, monitoring-based control, as indicated by the relation between CJs and control, was stronger for fourth than for second graders, *F* (1, 230) = 12.84, *p* < .001, η_p_^2^ = .05. Confirming our hypothesis of stronger monitoring-based control in the second measurement point, the results for open-ended questions indicated that the relationship between CJs and control was stronger at T_2_ compared to T_1_, *F* (1, 230) = 11.73, *p* = .001, η_p_^2^ = .05. For true-false questions the relation between monitoring and control did not differ between second and fourth graders, and between T_1_ and T_2_ (*p*s > .07).

##### Open-ended questions vs. true-false questions

We also investigated the difference in control accuracy between the two test formats. The difference between control accuracy (Gamma correlations between maintenance and performance) for open-ended and true-false questions was significant at T_1_, *F* (1, 206) = 22.43, *p* < .001, η_p_^2^ = .10, and T_2_, *F* (1, 231) = 35.75, *p* < .001, η_p_^2^ = .13; as visible in Table 3, control was more accurate for open-ended questions.

##### Stability

Table 2 shows the stability of control accuracy. For open-ended and true-false questions, stability for second and fourth graders was not significant (*p*s ≥ .90).

## Discussion

With the current study, we addressed elementary school children’s metacognition when taking a text comprehension test. The first aim was to investigate how monitoring and control develop during the elementary school years by examining developmental effects cross-sectionally and longitudinally. For this purpose, we tested second and fourth grade students twice within half a year with a text comprehension task for which children were asked to read texts, answer questions about the texts, give CJs for their answers, and decide whether they liked to maintain or withdraw each of their answers. The second aim was to examine how test format affects students’ accuracy of monitoring and control. To address this question, the text comprehension test consisted of open-ended and true-false questions.

Previous research on young elementary school children’s metacognition mainly used associative learning tasks with forced choice test formats, and individual testing of children. The present study extends this research by using an educationally relevant and more complex text comprehension task. In school, children often need to study texts and answer recall and recognition questions. Several studies investigated elementary school students’ metacognition during reading, and this research shows rather low accuracy of monitoring and control (e.g., De Bruin et al. 2011; Markman 1979). However, little is known about elementary school children’s monitoring and control when taking a text comprehension test; the present study aimed to address this. As question format influences monitoring and control during test taking in adults or older students (e.g., Ferrer et al. 2017; Pressley et al. 1990; Thiede and Dunlosky 1994), we included two different question formats to investigate whether this affects children’s metacognitive accuracy and the development of monitoring and control.

For open-ended questions, children demonstrated relatively well-developed metacognitive skills, reflected by higher confidence for correct answers compared to incorrect answers, and by maintaining more correct answers than incorrect answers. In line with our hypothesis that monitoring and control improve with age, discrimination between correct and incorrect answers was more pronounced for older than younger elementary school students. Particularly, fourth graders were less confident for their incorrect answers and maintained fewer incorrect answers than second graders. In fact, findings suggest that fourth graders rather cautiously controlled their performance, which not only led to accurately withdrawing more incorrect answers but also to slightly more frequent withdrawal of correct answers. However, due to the scoring scheme that included penalty for maintaining incorrect answers, fourth graders’ cautious controlling behavior implies a safe and efficient strategy, especially when considering the quality rather than the quantity of performance. Overall, these findings confirm previous research showing that metacognitive development is most pronounced for monitoring and controlling of incorrect, rather than correct answers (Krebs and Roebers 2010; Roderer and Roebers 2010). Moreover, both age groups improved their monitoring and control for incorrect answers within half a year.

There seem to be different reasons why children’s monitoring and control improve. Maturation of the prefrontal cortex has been proposed as an explanation for improvement in monitoring accuracy (Diamond 2002), and moreover, when getting older, children acquire more experiences with using learning strategies, receiving feedback, taking tests, and receiving metacognitive instructions by the teacher (Coffman et al. 2008; Grammer et al. 2013; Thiede et al. 2012; Van Loon and Roebers 2017). This presumably leads to a shift from basing one’s monitoring judgments and control decisions on motivational factors such as wishful thinking or effort that is put in a task (Schneider 1998), to cues that are more reflective of one’s performance, such as retrieval fluency or accessibility (Koriat and Ackerman 2010; Roebers et al. 2019; Van Loon et al. 2017). Using more valid cues consequently results in a more realistic evaluation of one’s performance, reflected in reduced overconfidence for incorrect answers. Older children’s improved monitoring accuracy, in turn, promotes effective control decisions, as control has shown to be related to monitoring (cf. Metcalfe and Finn 2013). Findings from our study highlight that this connection between monitoring and control increases over time.

Whereas both age groups successfully discriminated between correct and incorrect answers to open-ended questions, discriminating between correct and incorrect answers to true-false questions seemed more challenging for both age groups. That is, children were almost as confident for incorrect as correct answers, and maintained almost as many of their incorrect as their correct responses. Previous research showed that monitoring and controlling the quality of answers to recognition questions is challenging, even for adults.

One main reason for differences between question formats in monitoring and control accuracy seems to lie in differences of information generation processes during answering recall and recognition questions, and the cues that are provided through this processes (Yonelinas 2002). Future research could test if cue validity and cue utilization differ between open-ended and true-false questions. That is, if cues such as retrieval fluency are more valid for open-ended questions than true-false questions (e.g., stronger correlation between answer latency and correctness in open-ended vs. true-false questions), or if students more consistently use such cues when answering open-ended questions compared to true-false questions (e.g., stronger correlation between answer latency and CJs for open-ended questions than true-false questions).

Another reason for the lower metacognitive accuracy when answering true-false questions might have been the chance of guessing. Thiede and Dunlosky (1994) showed that the fewer the answer alternatives in a forced choice task, the higher the likelihood of guessing, and thus, the lower the accuracy of CJs. Because the recognition questions in the present study only had two alternatives (yes/no), students had the chance to pick the correct answer with a probability of 50%. Awareness of this probability might bias the confidence in one’s answers. Findings from Rohwer et al. (2012) indicate that even preschool children are aware of the probability of correctness when indicating certainty in their guessed answers.

Furthermore, the difference between monitoring and control for open-ended and true-false questions, could be due to (perceived) question difficulty. Thiede (1996) showed that subjects perceived recall questions as more difficult than recognition questions, even though objectively this was not the case. This difference in perceived question difficulty affected control, such that students spent more time studying for recall tests than for recognition tests. Thus, the feeling that open-ended questions may have been more difficult could have led children to become more cautious for their incorrect answers. Further, difficulty of questions seems to vary according to the level of inferences one has to draw from the text, with detail questions about the text being easier to answer than causal questions (Ferrer et al. 2017). The true-false questions in our study were detail questions, whereas open-ended questions contained detail as well as causal questions. Even though monitoring and control was somewhat more accurate for detail than for causal open-ended questions, monitoring and control for both types of open-ended questions, in comparison to true-false questions, was much more accurate. Hence, difficulty variations related to the level of inference making do not seem to be the main reason for the differences in metacognitive accuracy between open-ended and true-false questions.

Interestingly, children of both age groups improved their metacognitive accuracy for their answers to true-false questions at the second measurement point. That is, half a year after the first testing, they were less overconfident for, and maintained fewer of their incorrect answers. Thus, improvement over time for true-false questions paralleled the improvement for open-ended questions. The finding that children improved their metacognitive accuracy for true-false questions longitudinally but not cross-sectionally indicates that task familiarity is likely to have an impact on the accuracy of monitoring and control (cf. Schneider 1998).

Furthermore, we also addressed the stability of children’s metacognitive accuracy by investigating the correlations between the first and second measurement points. Even though performance was stable, stability of monitoring and control accuracy when discriminating between correct and incorrect answers was low for both age groups. This extends findings from Roebers and Spiess (2017) and Buratti et al. (2014) to monitoring and control of text comprehension. However, studies that included other measures of metacognitive accuracy, such as calibration or overconfidence, show higher levels of stability (Buratti et al. 2014; Jonsson and Allwood 2003; Roebers and Spiess 2017; Stankov and Crawford 1996). Further research could investigate whether stability in monitoring and control of text comprehension varies as a function of measurement.

### Limitations

Despite of the merits, our study also has some limitations. First, as shown in most studies comparing recall and recognition questions (e.g., Thiede and Dunlosky 1994), performance was higher for true-false questions than for open-ended questions, and this may result in limitations when comparing metacognition related to these two test formats. However, gamma correlations, which are considered independent of test performance (Nelson 1984), support our conclusions about the differences in monitoring and control as an effect of test format, and this may indicate that test factors influence metacognition beyond performance.

Furthermore, second graders had more correct answers and fewer omissions when answering open-ended questions at T_2_ than T_1_. A possible explanation for this improvement in performance and decrease in omissions is that students improved their comprehension skills and were thus better able to answer questions. This could, in turn, have had an impact on metacognitive accuracy. Literature indicates that skills related to the first order task (e.g., reading, spelling etc.) affects metacognitive accuracy in this task (e.g., Roebers and Spiess 2017; Vidal-Abarca et al. 2010).

A further limitation may be that texts were presented on screen and not in print. Some studies indicate that text comprehension might differ between reading on screen versus on paper (Mangen et al. 2013), whereas others obtained similar test results for reading comprehension tasks on screen and paper (Ferrer et al. 2017). Possibly, findings from this research are not directly generalizable to activities in which children have to study printed materials. However, it has to be noted that nowadays, students more and more need to acquire their knowledge by reading on computers or tablets instead of using more traditional paper print sources (Cartelli 2012).

Furthermore, even though correlations between performance on open-ended and true-false questions were moderate to high, indicating that both test types actually measured text comprehension, internal consistency of the open-ended as well as true-false questions was rather low, especially for true-false questions. Such low internal consistency has also been observed in other research assessing text comprehension for different texts (e.g., Engelen et al. 2018). Because children read six different texts, it could be that each child’s understanding of the different texts varied, thus resulting in higher performance on questions for certain texts and lower performance for other texts. Because we were primarily interested in children’s monitoring processes related to text comprehension, we aimed to have variance in text topics and test questions. That is, the main purpose of this study was to include items with various difficulty levels, as it is mostly the case in school tests, and to investigate the monitoring processes that come along. Although this might have negatively affected internal consistency of the tests itself, it provided informative insights into the metacognitive processes, and to what extent children were able to differentiate between well-learned and less-learned texts.

Of course, different factors such as brain maturation, environmental influences and familiarity with the task seem plausible reasons for the observed improvements over time. However, the design of our study does not allow to draw conclusions about the specific impact of each of these factors. Future research could aim to disentangle “natural” developmental effects (brain maturation and environmental) from familiarity with the specific task by including a control group that completes the task only at the second measurement point.

### Conclusion and implications

Taken together, our study sheds light on elementary school children’s metacognitive skills when learning from texts. Findings show that the developmental progression of monitoring and control, mainly found in paired associate tasks, also applies to a more educationally valid text-learning task. However, this age-dependent progression was only found when monitoring and controlling open-ended questions. The present study highlights the discrepancy in metacognitive accuracy between open-ended and true-false questions; metacognitive accuracy was considerably higher for open-ended questions. Additionally, it also demonstrated that children’s monitoring and control accuracy varies over time, rather than being a stable characteristic.

Investigating the conditions under which metacognition is inaccurate can reveal important insights that can be applied in practice. Findings from intervention studies showed that the promotion of self-regulated learning, which includes metacognition, has an impact on students’ academic performance and metacognitive strategy use (Dignath et al. 2008; Kistner et al. 2010). This emphasizes the importance of teachers focusing on monitoring and control during their lessons. Our findings indicate that when aiming to improve children’s monitoring and control through taking self-tests, teachers should use open-ended questions instead of recognition questions such as true-false questions, because children seem to be able to learn and improve the most when using this type of questions. When using true-false questions, teachers should be aware that this format will make monitoring and control challenging for elementary school children, regardless of their age.

## Electronic supplementary material

ESM 1 (PDF 500 kb)

## Footnotes

1 The study phase was followed by judgments of learning (indicating how confident students were that they will be able to answer the questions for each text) and a restudy wish (indicating which texts they would restudy—no real restudy happened). Judgments of learning and indication of restudy wish were not included in our study as our hypotheses focused on metacognition during test taking, and therefore metacognition before test taking goes beyond the scope of this research. However, for interested readers we added the values for monitoring and control accuracy before test taking in the Appendix (Appendix B).
