# Supplementary material for: Development of Children’s monitoring and control when learning from texts: effects of age and test format
Source: Metacogn Learn. 2019 Sep 7;15(1):3–27. doi: 10.1007/s11409-019-09208-5 (PMC7089689; doi:10.1007/s11409-019-09208-5)
Supplement: Supplementary file 1 — (PDF 500 kb) [file 11409_2019_9208_MOESM1_ESM.pdf]

Appendix A

Example of a text-questions set for second and fourth graders

Topic: Chewing gum

Version for second graders:

Already in Roman times chewing gum was chewed. However, the first chewing gums were without flavor. Although chewing gum is a strange material, there is no reason not to swallow it.

Chewing gum is made of synthetic material and can't be digested. It leaves the human body unaltered. This means that a chewing gum doesn't stay in the body for a long time. No chewing gum clog was ever found in the stomach of a human being, even though a lot of children swallow their chewing gums.

Open-ended questions:

What happens with a chewing gum in your stomach? *(Correct response: it leaves the body unaltered/it won't be digested)*

How did the first chewing gums taste? *(Correct response: no taste/flavor)*

True-false statement questions:

Already the Romans chewed gum. *(True)*

Chewing gums clots are often found in the stomach of children. *(False)*

<u>Version for fourth graders:</u>

Already in Roman and Egyptian times chewing gum was chewed. The first chewing gum manufacturer was the American John Curtis Jackson in 1848. The chewing gums were without flavour. It was only after 1975 when flavors like licorice or peppermint were added to the chewing mass.

Even though chewing gum is a strange material, there is no medical reason not to swallow it. Chewing gum is made of a synthetic material called Polyvinyl acetate. This synthetic material leaves the human body unaltered. This means that a chewing gum doesn't stay in the body for a long time. No chewing gum clog was ever found in the stomach of a human being, even though a lot of children swallow their chewing gums.

Open-ended questions:

What happens when you swallow a chewing gum? *(Correct answer: it leaves the body unaltered/it won't be digested)*

What material are chewing gums made of*? (Correct answer: synthetic material/Polyvinyl acetate)*

True-false statement questions:

Already the Romans and Egyptians chewed gum. *(True)*

It takes years until a swallowed chewing gum is digested by the body. *(False)*

Appendix B

Gamma Correlations of Prediction Measures (JOL/Restudy Wish) With Test Performance

	2nd grade		4th grade	
	T_1	T_2	T_1	T_2
Judgments of Learning	-.01 *(.62)*	.08 *(.52)*	.00 *(.53)*	.21 *(.58)***
Restudy Wishes	0.00 *(.75)*	-.05 *(.70)*	-.01 *(.74)*	-.18 *(.72)**

Note. Standard deviations in parentheses. Gamma correlations between an individual's Judgment of Learnings and performance, and between an individual's Restudy Wishes and performance, computed across the set of six texts. In the context of Restudy Wishes, a stronger negative correlation indicates more effective control. Significance level indicates if value differs from zero.

* $p < .05$. ** $p < .001$.
